# Lipidome profile predictive of disease evolution and activity in rheumatoid arthritis

**DOI:** 10.1038/s12276-022-00725-z

**Published:** 2022-02-15

**Authors:** Jung Hee Koh, Sang Jun Yoon, Mina Kim, Seonghun Cho, Johan Lim, Youngjae Park, Hyun-Sook Kim, Sung Won Kwon, Wan-Uk Kim

**Affiliations:** 1 Division of Rheumatology, Department of Internal Medicine, the Catholic University of Korea, Seoul, 06591 Republic of Korea; 2 Center for Integrative Rheumatoid Transcriptomics and Dynamics, the Catholic University of Korea, Seoul, 06591 Republic of Korea; 3 College of Pharmacy, Seoul National University, Seoul, 08826 Republic of Korea; 4 Department of Statistics, Seoul National University, Seoul, 08826 Republic of Korea; 5 Department of Internal Medicine, Soonchunhyang University College of Medicine, Seoul, 04401 Republic of Korea

**Keywords:** Rheumatoid arthritis, Metabolomics

## Abstract

Lipid mediators are crucial for the pathogenesis of rheumatoid arthritis (RA); however, global analyses have not been undertaken to systematically define the lipidome underlying the dynamics of disease evolution, activation, and resolution. Here, we performed untargeted lipidomics analysis of synovial fluid and serum from RA patients at different disease activities and clinical phases (preclinical phase to active phase to sustained remission). We found that the lipidome profile in RA joint fluid was severely perturbed and that this correlated with the extent of inflammation and severity of synovitis on ultrasonography. The serum lipidome profile of active RA, albeit less prominent than the synovial lipidome, was also distinguishable from that of RA in the sustained remission phase and from that of noninflammatory osteoarthritis. Of note, the serum lipidome profile at the preclinical phase of RA closely mimicked that of active RA. Specifically, alterations in a set of lysophosphatidylcholine, phosphatidylcholine, ether-linked phosphatidylethanolamine, and sphingomyelin subclasses correlated with RA activity, reflecting treatment responses to anti-rheumatic drugs when monitored serially. Collectively, these results suggest that analysis of lipidome profiles is useful for identifying biomarker candidates that predict the evolution of preclinical to definitive RA and could facilitate the assessment of disease activity and treatment outcomes.

## Rheumatoid arthritis: Changes in lipid profile mark disease activity

The profile of lipid metabolites changes according to the different phases of rheumatoid arthritis (RA) activity, providing potential biomarkers for tracking and treating the disease. Lipid metabolites play diverse roles in the body, including acting as signaling molecules for inflammation. Wan-Uk Kim (Catholic University of Korea, Seoul), Sung Won Kwon (Seoul National University), and co-workers across South Korea analyzed the lipid profiles (or ‘lipidomes’) of joint fluid and serum samples were taken from 79 patients (42 from active RA, 19 from sustained remission, 18 from preclinical RA) at the preclinical, active, and remission phases of RA. The lipidome of joint fluid was significantly disrupted both in preclinical and active RA, and the extent of disruption matched the severity of disease. There were marked differences in the serum lipidome between active and remission phases. Monitoring lipidome profiles could prove invaluable for early diagnosis, and may inform future precision therapeutics.

## Introduction

A better understanding of the pathogenesis of rheumatoid arthritis (RA) has led to a marked improvement in patient management over the past three decades; inexorable joint destruction and disability have become infrequent, and sustained remission is a realistic target of treatment^1–3^. Although early diagnosis and early initiation of disease modifying anti-rheumatic drug (DMARD) therapy are key components of the treatment strategy, the definition of ‘early RA’ is not clear. Conventionally, it refers to the initial phase, when arthritis is detected clinically^4^. Rheumatoid factor (RF) and anti-citrullinated protein antibodies (ACPAs) are autoantibodies representative of RA; these antibodies precede the clinical manifestations of RA by a median of 4.5 years^5^. To date, these two autoantibodies are the only available biomarkers for early RA^6,7^. However, approximately half of patients with positive RF and/or ACPA do not develop RA^5,8^.

When implementing treat-to-target strategies, the assessment of treatment response is an important component^9^. Tracking changes in disease activity using composite disease activity indices (such as the disease activity score in 28 joints [DAS28]) before and after treatment is valid^10^. Due to the intricacy of those indices, the erythrocyte sedimentation rate (ESR) and C-reactive protein (CRP) levels are used practically to estimate disease activity in most real-world clinics. However, ESR and CRP show limited specificity and sensitivity as biomarkers for monitoring RA disease activity, especially in patients taking cytokine blocking agents^11,12^; therefore, there is great interest in identifying alternative and reliable biomarkers that can improve the assessment of treatment responses.

Lipids are essential metabolites that act as energy sources, membrane constituents, and signaling molecules^13^. Importantly, lipids act as signaling molecules for inflammation; for example, eicosanoids precede the production of cytokines and chemokines, and phosphoinositides serve as lipid-derived second messengers^14^. Although recent studies have investigated lipid mediators generated by cyclooxygenases or lipoxygenases during the preclinical stage of RA^15,16^, no study has taken an unbiased and systematic approach to determining the lipidome signatures underlying preclinical RA, dynamic changes in lipidome profiles according to RA disease activity, and treatment responses. Lipidomics, the study of the total lipid content of cells or biofluids using the principles and techniques of analytical chemistry^17^, may provide a direct readout of candidate lipid biomarkers of RA progression and resolution and facilitate the assessment of treatment responses.

In this study, we attempted a global and systematic integrated analysis of changes in the lipidome profile at different phases of RA, from the preclinical phase to the active phase to the sustained remission phase. We show that the lipidome profile in the arthritic joint fluids of RA patients is markedly perturbed and that the level of perturbation correlates with the extent of inflammation and severity of synovitis. We also show that the serum lipidome profile is significantly altered in individuals at the preclinical phase during which the ESR and CRP levels are normal. Moreover, the lipid profile sensitively reflects RA activity and the treatment response to DMARDs. Collectively, analysis of lipidome profiles may be useful for identifying candidate biomarkers that predict progression of preclinical to clinically overt RA, as well improving assessment of disease activity and treatment outcomes.

## Materials and methods

### Study participants

The study participants were enrolled in the Center for Integrative Rheumatoid Transcriptomics and Dynamics (CIRAD) cohort, a prospective cohort of RA patients at Seoul St. Mary’s Hospital initiated in 2015. The cohort comprises patients with RA, preclinical RA, and osteoarthritis (OA). All participants with RA fulfilled the 2010 American College of Rheumatology (ACR)/European League Against Rheumatism (EULAR) RA classification criteria^6^. Preclinical RA was defined as a phase at which an individual with arthralgia shows an increase in RF and/or ACPA of more than three times the normal limit while not yet fulfilling the 2010 ACR/EULAR classification criteria^4^. Those patients were followed-up to observe whether they developed definite RA by March 2021. Clinical and demographic data were obtained from the CIRAD cohort, which was monitored regularly. Serum was collected from each participant at the time of enrollment and stored at −80 °C for subsequent analysis. For patients with RA, serum was also collected and stored every 6 months. SF was also collected when it was aspirated and centrifuged immediately, and the supernatant was stored at −80 °C. WBC counts (/μL) in the SF and the synovitis score measured by ultrasound examination^18^ were determined at the time of SF aspiration.

Considering the potential impact on the lipidome profile, patients taking lipid-lowering agents were excluded from analysis. To investigate lipid changes according to treatment outcome, 42 patients with moderate-to-high disease activity (DAS28 > 3.2) at baseline were selected. For these patients, serum samples were obtained at baseline and at 6 months after treatment with DMARDs. To investigate differences in lipidome profiles according to disease phase, 19 RA patients in sustained remission, defined as patients with DAS28 ≤ 2.6 measured at three consecutive times over 12 months, were selected. In addition, 18 preclinical RA patients not receiving DMARDs or lipid-lowering agents were selected. Age- and sex-matched OA patients were selected as controls. Furthermore, 71 RA-SF and 31 OA-SF samples from patients who did not use lipid-lowering agents were analyzed. To validate the association between RA activity and altered lipidome profiles in sera, 61 samples obtained on different occasions were selected from patients with active RA or sustained remission, and their lipidomes were measured independently in a different batch.

### Ethics

The study was approved by the institutional review board of Seoul St. Mary’s Hospital, the Catholic University of Korea (KC16SISI0632). All study participants provided written informed consent.

### Sonographic evaluation of joints for synovitis severity

Musculoskeletal ultrasonography was performed for RA patients from whom SF samples were obtained. The synovitis score was determined using the grayscale and power-Doppler grades. The grayscale score (range, 0–3) was defined as the degree of synovial hypertrophy in the joints, as follows: grade 0 = no synovial hypertrophy, grade 1 = minimal synovial hypertrophy, grade 2 = moderate synovial hypertrophy, and grade 3 = severe synovial hypertrophy^19^. The power Doppler score (range, 0–3) was assessed according to the extent of vascularity within the synovium of joints as follows: grade 0 = no Doppler activity, grade 1 = minimal Doppler activity, grade 2 = moderate Doppler activity (≤50% of the background synovium), and grade 3 = severe Doppler activity (>50% of the background synovium)^18,19^. The severity of synovitis was determined using the EULAR-OMERACT combined score system: mild synovitis (synovitis score 0 or 1) and moderate-to-severe synovitis (synovitis score 2 or 3)^19^.

### Preparation of serum and SF samples

The *tert*-methyl butyl ether (MTBE) method was used with modification to extract lipids from serum and SF samples^20^. During the extraction step, two internal standard (IS) mixtures and water were used. LPC (17:0), PC (10:0/10:0), PE (10:0/10:0), and SM (18:1/17:0) were prepared in methanol (Mix1). LPE (17:1), TG (17:0/17:1/17:0, d_5_), DG (12:0/12:0), and Cer (18:1/17:0) were prepared in MTBE (Mix2). First, a 50 μL aliquot of each serum or SF sample was placed into a microcentrifuge tube, and 300 μL Mix1 was added and vortexed. Next, the tubes were incubated in a Thermo shaker (0 °C, 1500 rpm, 1 h) after the addition of 1 mL of Mix2. Next, 250 μL of water was put into the tubes, which were then vortexed for 1 min and centrifuged (16,000 rcf for 10 min). Finally, the upper hydrophobic layer was separated and filtered through a hydrophobic syringe filter unit. The filtrate was then evaporated under nitrogen gas to obtain fully dried lipid extracts. For instrumental analysis, the extracts were reconstituted in a 100 μL methanol/toluene mixture (9:1 v/v) shortly before analysis.

### UPLC-Q-ToF MS-based untargeted lipidomics analysis

Lipids were separated on an ultra-performance liquid chromatography (UPLC) system equipped with a C18 column (2.1×100 mm, 1.7 μm) connected to a guard column (2.1×5 mm, 1.7 μm). Both mobile phases (A: 40:60 (v/v) water:acetonitrile and B: 10:90 (v/v) acetonitrile:isopropanol) contained 10 mM ammonium formate and 0.1% formic acid. For data acquisition, an Agilent 6530 quadrupole time-of-flight mass spectrometer (Q-ToF MS) was operated in ESI-positive mode, and an MS/MS data-dependent acquisition mode was applied. The gradient conditions and MS conditions were as described previously^21^. Serum and SF samples were analyzed after division into three and two batches, respectively, and each batch comprised a random sequence of samples.

### Data preprocessing and lipid identification

Raw data were imported into MS-DIAL ver 4.38 after format conversion for a series of data preprocessing from data collection to alignment^22^. Batch effects were removed by a locally estimated scatterplot smoothing (LOESS) algorithm^23^. After peak annotation, unreliable lipids were excluded when the relative standard deviation (RSD, %) of the quality control (QC) samples was >30%. Annotated lipids were identified putatively based on matching precursor ion *m/z* values and the product ion pattern of the data to the LipidBlast database in MS-DIAL. Next, the identified lipid list was confirmed by an in-house lipid library, including retention times, to improve the confidence in lipid identification as metabolomics standards initiative (MSI) level 1.

### Lipid ontology enrichment analysis

Ontology analysis based on all identified lipids was performed by LION^24,25^. The list of identified lipids was inputted, along with the normalized intensity of each lipid, into software to facilitate the export of an enrichment table and a PCA heatmap. Lipids with no matches in the LION database were excluded prior to analysis.

### Correlation analysis

Correlation analysis was performed using the *Hmics* package in R. Pearson’s correlation analysis of the correlation between normalized lipid intensity and clinical indicator analysis was conducted using the *rcorr* function. *P* values from Pearson’s correlation analysis were used to test the significance of the correlation coefficient. Samples in which clinical indicators were not measured were excluded.

### Biomarker analysis

The biomarker candidates showing |*r*| ≻0.5 in the orthogonal projections to latent structures-discriminant analysis (OPLS-DA) model, or FDR values <0.25, were selected. The AUC of the ROC curve was calculated using the random forest algorithm. Average accuracy was calculated based on 100 cross-validations.

### Statistical analysis

All statistical analyses were performed using MetaboAnalyst 5.0^26^. Every lipid feature was normalized according to the median intensity of each sample. Lipids with >50% missing values were excluded, and the remaining missing values were replaced by the median intensity value for the lipid feature. Finally, log transformation and Pareto scaling were performed. A *t* test was performed using the Benjamini–Hochberg procedure to acquire the FDR, and a paired *t* test was used to compare groups before and after treatment. *P* values <0.05 and FDR values <0.25 were considered significant. For comparison of more than three groups, one-way ANOVA with Tukey’s HSD was used to identify significant differences. Suspected outliers were excluded by a cautious investigation based on comprehensive interpretation using a heatmap, PCA, and outlier suggestion by the random forest algorithm. Two different multivariate statistical analysis models, unsupervised and supervised, were applied to discriminate the groups (unsupervised, PCA; supervised, PLS-DA). PLS-DA models were cross-validated using leave-one-out cross validation (LOOCV), and the *Q*^2^ value was used to estimate overfitting of the model. Lipids with a VIP score value >1 were defined as crucial for discriminating the groups. Multivariate exploratory ROC analysis was performed by Monte Carlo cross validation (MCCV) using balanced subsampling (2/3 of samples were used to estimate feature importance, and 1/3 were used for model validation) and classified by random forests. Regularized Hotelling’s T^2^ (RHT) test was applied to obtain RHT statistics, and *p* values based on the null distribution (distribution of the RHT statistic under the null hypothesis, assuming insignificant differences between two groups) were approximated by resampling.

## Results

### Lipidome profiles in the synovial fluid of RA patients are altered significantly

To investigate the effect of inflammation on the lipidome profile, SF samples from patients with RA (RA-SF) were analyzed as two separate groups: samples with a white blood cell (WBC) count <3000/μL (leukocyte-poor RA-SF) and samples with a WBC count ≥3000/μL (leukocyte-rich RA-SF). There was no difference in the demographic data between the OA-SF, leukocyte-rich RA-SF, and leukocyte-poor RA-SF groups (Supplementary Table 1). In the RA patients, the leukocyte-rich RA-SF group showed higher acute phase reactant levels than the leukocyte-poor RA-SF group.

**Fig. 1:**
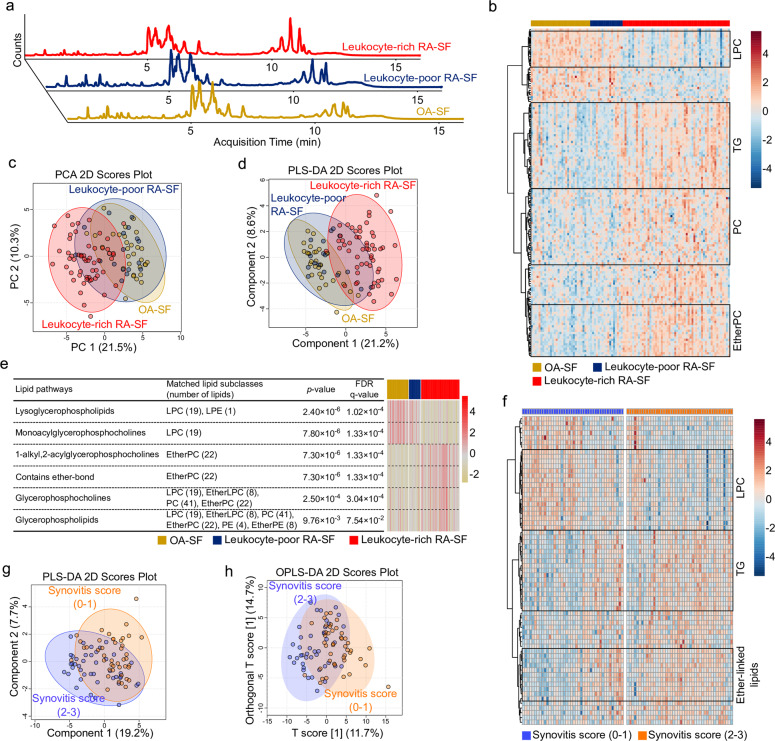
The lipidome profile and its associated pathways in synovial fluid from RA patients. **a** Representative LC-MS/MS chromatogram of lipid extracts belonging to three different synovial fluid (SF) groups (osteoarthritis [OA]-SF, leukocyte-poor RA-SF, and leukocyte-rich RA-SF). **b** Heatmap of normalized intensity demonstrating significant differences among lipids in the three groups. The lipid subclasses in the heatmap are as follows: lysophosphatidylcholine (LPC), triacylglycerol (TG), phosphatidylcholine (PC), and ether-linked phosphatidylcholine (EtherPC). **c** PCA 2D score plots and **d** PLS-DA 2D score plots based on lipidome profiles. **e** Heatmap of lipid-related pathways that were significantly different among the three groups. The normalized scale of the six pathways in each sample is indicated from red (high) to yellow (low). **f** Heatmap of lipids that differ significantly according to the synovitis score measured by ultrasound. A synovitis score of 0 or 1 denotes mild synovitis, and a synovitis score of 2 or 3 denotes moderate-to-severe synovitis. **g** PLS-DA 2D score plot and **h** OPLS-DA 2D score plot derived from the models established by assignment of mild synovitis and moderate-to-severe synovitis based on the synovial lipidome. Abbreviations: EtherLPC ether-linked lysophosphatidylcholine, EtherPE ether-linked phosphatidylethanolamine, LPE lysophosphatidylethanolamine, OPLS-DA orthogonal partial least squares discriminant analysis, PCA principal component analysis, PLS-DA partial least-squares discriminant analysis.

Representative chromatograms from the leukocyte-rich RA-SF, leukocyte-poor RA-SF, and OA-SF groups are shown in Fig. 1a. Following sequential data preprocessing, we identified 205 synovial lipidomes belonging to 13 lipid subclasses; we then conducted one-way analysis of variance (ANOVA) followed by Tukey’s honestly significant difference (HSD) test to identify significant differences in lipidome expression among these groups (Table 1). We found significant intergroup differences (based on Tukey’s HSD) in 144 synovial lipids: 14 between leukocyte-poor RA-SF and OA-SF, 124 between leukocyte-rich and leukocyte-poor RA-SF, and 143 between leukocyte-rich RA-SF and OA-SF (Table 1**;** *a detailed list of lipids is available at Online Supplemental file 1*). Overall, lipidome expression patterns in the leukocyte-rich RA-SF groups were markedly different from those in the other two groups, whereas there was no difference between the OA-SF and leukocyte-poor RA-SF groups (Fig. 1b). In particular, among the main lipid subclasses, lysophosphatidylcholine (LPC) was markedly reduced, whereas phosphatidylcholine (PC), ether-linked PC (EtherPC), triacylglycerol (TG), and sphingomyelin (SM) were markedly increased in the leukocyte-rich RA-SF group (Fig. 1b).

**Table 1:** Number of significant synovial lipids belonging to each lipid subclass (based on multiple comparisons).

Lipid subclass	Total identifiedlipids (*n*)	Significantlipids (*n*)^a^	Sig./total(%)^b^	Leukocyte-poor RA-SF-OA-SF (*n*)	/sig. lipid (%)	Leukocyte-rich RA-SFLeukocyte-poor RA-SF (*n*)	/sig. lipid (%)	Leukocyte-rich RA-SF-OA-SF (*n*)	/sig. lipid (%)
CAR	14	4	28.6	-	-	4	100.0	4	100.0
Cer	4	4	100.0	-	-	3	75.0	4	100.0
SM	15	12	80.0	-	-	12	100.0	12	100.0
LPC	19	17	89.5	4	23.5	16	94.1	17	100.0
EtherLPC	8	6	75.0	1	16.7	6	100.0	6	100.0
LPE	1	1	100.0	-	-	1	100.0	1	100.0
EtherLPE	1	1	100.0	-	-	1	100.0	1	100.0
PC	41	25	61.0	-	-	20	80.0	25	100.0
EtherPC	22	21	95.5	2	9.5	20	95.2	21	100.0
PE	4	3	75.0	2	66.7	1	33.3	3	100.0
EtherPE	8	5	62.5	-	-	3	60.0	5	100.0
DG	1	1	100.0	1	100.0	-	-	1	-
TG	67	44	65.7	4	9.1	37	84.1	43	97.7
Total	205	144		14		124		143	

The number and percentage of significant lipids/total identified lipids belonging to each subclass are shown.

*CAR* acyl carnitine, *Cer* ceramide, *DG* diacylglycerol, *EtherLPC* ether-linked LPC, *EtherLPE* ether-linked LPE, *EtherPC* ether-linked PC, *EtherPE* ether-linked PE, *LPC* lysophosphatidylcholine, *LPE* lysophosphatidylethanolamine, *PC* phosphatidylcholine, *SM* sphingomyelin, *TG* triacylglycerol.

^a^Differentially expressed lipids were identified by one-way ANOVA and Tukey’s HSD test.

^b^The percentage of significant lipids (sig.) divided by the total number of identified lipids.

Two-dimensional (2D) score plots of principal component analysis (PCA) and partial least squares-discriminant analysis (PLS-DA) demonstrated that lipidome profiles also discriminated leukocyte-rich RA-SF from OA-SF opand leukocyte-poor RA-SF; however, the lipidome profiles of OA-SF and leukocyte-poor RA-SF almost overlapped (Fig. 1c, d). The optimal *Q*^*2*^ for the PLS-DA model was 0.576. In addition, the variable importance in the projection (VIP) score value for 88 synovial lipids was >1; these lipids were defined as important for discriminating the groups in the PLS-DA model (*a detailed list of lipids is available at Online Supplemental file 1*).

The normalized intensity of the identified synovial lipids was used to perform lipid ontology enrichment analysis to estimate lipid metabolism in the inflammatory microenvironment of RA joints. Six lipid-related pathways were differentially expressed (Fig. 1e; the list of individual lipids matched to each lipid pathway is shown in Supplementary Table 2). ‘Lysoglycerophospholipids’ and ‘monoacylglycerophosphocholines’ were downregulated in leukocyte-rich RA-SF compared with OA-SF and leukocyte-poor RA-SF. In contrast, ether-lipid metabolism (‘1-alkyl,2-acylglycerophosphocholines’ and ‘contains ether bonds’) and glycerophospholipid metabolism (‘glycerophosphocholines’ and ‘glycerophospholipids’) were overexpressed in leukocyte-rich RA-SF compared with the other two groups. The *p* values, false discovery rate (FDR), and a matched lipid list of six significant lipid-related pathways are shown in Fig. 1e.

### Correlation of synovial lipidome profiles with inflammatory activity and synovitis severity

To investigate changes in synovial lipidome profiles triggered by the local inflammatory microenvironment in RA, we merged the three different SF groups to form a two-comparison set. First, we used a *t* test and PLS-DA data to identify/extract lipids differentially expressed between OA-SF and RA-SF (leukocyte-rich RA-SF plus leukocyte-poor RA-SF) to estimate the effect of RA itself on the lipidome profile. At the same time, leukocyte-rich SF (leukocyte-rich RA-SF) and leukocyte-poor SF (leukocyte-poor RA-SF plus OA-SF) were also assessed using a *t* test and PLS-DA to identify lipid alterations due to leukocytosis (Supplementary Fig. 1). The results showed that 135 synovial lipids were associated both with RA and with the extent of leukocytosis; in addition, five lipids were associated with RA alone, and 15 were associated with leukocytosis alone (Supplementary Fig. 1; *the list of significantly different lipids is available at Online Supplemental file 2*).

Next, we investigated which types of specific lipids best represent synovitis severity, as determined by ultrasonography (which reflects histological synovitis)^27,28^. The severity of synovitis was assessed by ultrasonography at the time of SF aspiration. The levels of 65 lipids were significantly different between subgroups showing mild synovitis (synovitis score 0 or 1) and moderate-to-severe synovitis (synovitis score 2 or 3) (Fig. 1f; *a detailed list of these lipids is available at Online Supplemental file 3*). To assess whether the identified lipids were differentially expressed in patients with mild synovitis or moderate-to-severe synovitis, we used PLS-DA and OPLS-DA. We observed two distinct clusters representing each of these subgroups (Fig. 1g, h). Similar to the lipidome signature noted in leukocyte-poor versus leukocyte-rich SF, the levels of synovial LPC, ether-linked LPC (EtherLPC), LPE 16:1, and acylcarnitine (CAR) were lower in those with moderate-to-severe synovitis than in those with mild synovitis. In contrast, there was a marked increase in synovial PC, EtherPC, and TG in those with moderate-to-severe synovitis (Fig. 1f).

**Fig. 2:**
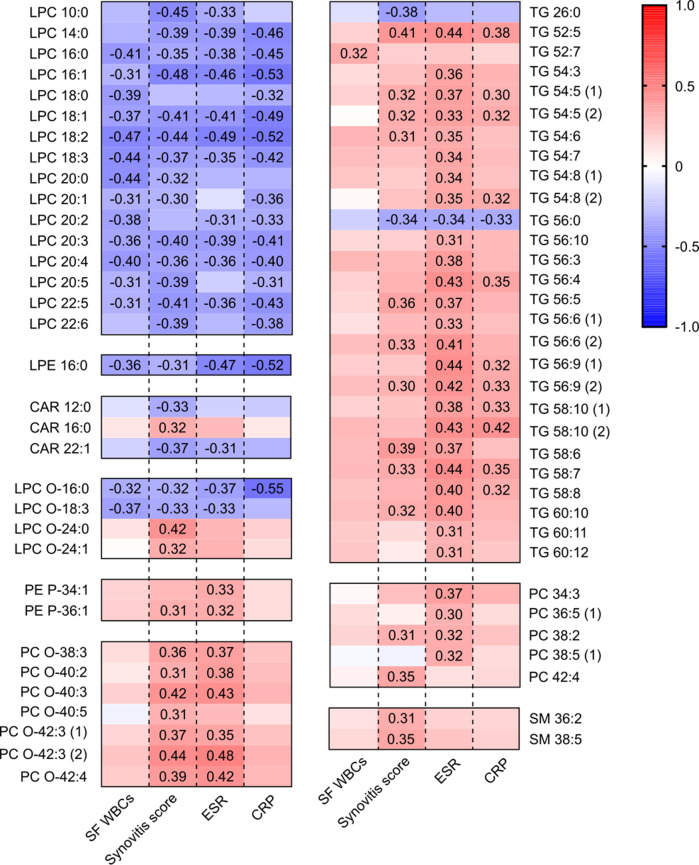
Pearson’s correlation analysis of lipids and laboratory and clinical parameters. Exact values for the correlation coefficients are depicted in a heatmap when the absolute values of the correlation coefficients are ≥0.3 (i.e., the *p* values for the correlation coefficients were <0.05). Red indicates a positive correlation, and blue indicates a negative correlation with acute phase reactants (erythrocyte sedimentation rate (ESR) and C-reactive protein (CRP)) and synovial inflammatory parameters (the synovitis score measured by ultrasonography and white blood cell counts in synovial fluid). Abbreviations: CAR acylcarnitine, LPC lysophosphatidylcholine, LPC O- ether-linked LPC, LPE lysophosphatidylethanolamine, PC phosphatidylcholine, PC O- ether-linked phosphatidylcholine, PE P- ether-linked phosphatidylethanolamine, SM sphingomyelin, TG triacylglycerol.

To further characterize the association between the synovial lipidome and systemic inflammatory activity in RA, we compared the laboratory and sonographic parameters with the lipidome profiles. Correlation analysis demonstrated that among the 135 synovial lipids that were considered sensitive to both RA itself and leukocytosis, 67 correlated well with the blood ESR, CRP levels, and synovitis score (correlation coefficient (|*r*|) >0.3) (Fig. 2). Specifically, synovial LPC and lyshophosphatidylethanolamine (LPE) 16:0 showed a negative correlation with the ESR, CRP levels, WBC counts in the SF, and synovitis score. In contrast, ether-linked phospholipids, including EtherPC and ether-linked phosphatidylethanolamine (EtherPE), and TG correlated positively with the synovitis score and the ESR. Synovial sphingomyelin (SM) correlated positively with the synovitis score but not with the ESR or CRP (Fig. 2). These results indicate that synovial LPC and LPE 16:0 were negatively associated with both systemic and local inflammatory activity in RA, whereas synovial EtherPC, EtherPE, and TG were positively associated with these parameters. Additionally, synovial SM seems to be associated only with synovitis severity.

To identify synovial lipid biomarker candidates that distinguish synovitis severity, we performed multivariate exploratory receiver operating characteristic (ROC) curve analysis using MCCV and balanced subsampling (Supplementary Fig. 2a). The area under the ROC curve (AUC) and the predictive accuracy of 50 important synovial lipids provided the greatest discrimination between moderate-to-severe synovitis and mild synovitis (Supplementary Fig. 2b). However, 15 synovial lipids were chosen for practical and clinical applicability given that every lipid except PC O-40:8 was differentially expressed in moderate-to-severe synovitis (Supplementary Fig. 2a) and that the 15 lipids showed AUCs and predictive accuracy similar to those of all 50 synovial lipids (Supplementary Fig. 2b, c).

Taken together, these results indicate that the synovial lipidome signature is significantly altered in RA joints and that it is representative of local and systemic inflammatory activity as well as the pathologic severity of synovitis; this suggests that some lipids could be a diagnostic marker for RA and, presumably, the level of RA activity.

### Serum lipidome profiles according to RA phase and activity

Our next goal was to identify serum lipid biomarkers that sensitively reflect RA activity and treatment outcomes in RA patients. To monitor alterations in the expression of the serum lipidome, we performed mass spectrometry-based lipidomics using serum samples from 18 patients with preclinical RA, 42 patients with active RA (DAS28 > 3.2 at baseline) and with a 6-month follow-up monitoring period, and 19 patients in sustained remission (DAS28 ≤ 2.6 measured consecutively over 12 months); 49 patients with OA were included as a non-RA control. The baseline demographics of the four groups were similar; the exceptions were disease activity-related parameters such as the ESR, CRP, and DAS28 (Table 2).

**Table 2:** Baseline characteristics of the serum donors.

	Preclinical RA (*n* = 18)	Active RA (*n* = 42)	RA in SR (*n* = 19)	OA (*n* = 49)	*P* value
Female, *n* (%)	15 (83.3%)	37 (88.1%)	16 (84.2%)	43 (87.8%)	0.941
Age (years)	52.1 ± 9.8	56.6 ± 12.7	55.5 ± 9.2	54.7 ± 11.2	0.836
BMI, kg/m^2^	23.2 ± 2.4	22.7 ± 2.8	22.1 ± 2.4	23.1 ± 3.3	0.945
RA duration, yr	-	8.9 ± 8.7	9.0 ± 9.9	-	0.955
RF-positive, *n* (%)	15/18 (83.3)	37/42 (88.1)	13/19 (68.4)	-	0.164
ACPA-positive, n (%)	14/18 (77.8)	31/35 (88.6)	11/16 (68.8)	-	0.118
Laboratory Findings					
ESR, mm/h	12.2 ± 13.1	28.4 ± 19.2	8.0 ± 6.9	7.6 ± 5.6	<0.001
CRP, mg/dl	0.2 ± 0.3	1.1 ± 1.1	0.2 ± 0.2	0.2 ± 0.5	0.004
Albumin, g/L	4.4 ± 0.2	4.1 ± 0.4	4.2 ± 0.2	4.4 ± 0.2	0.633
Hemoglobin, g/L	14.4 ± 6.2	12.5 ± 1.1	12.9 ± 1.2	13.5 ± 1.2	0.846
DAS28	-	4.7 ± 1.0	1.7 ± 0.7	-	<0.001
MTX, *n* (%)	-	26 (61.9%)	15 (78.9%)	-	0.309
HCQ, *n* (%)	-	14 (33.3%)	10 (52.6%)	-	0.104
SSZ, *n* (%)	-	5 (11.9%)	3 (15.8%)	-	0.705
LEF, *n* (%)	-	14 (33.3%)	10 (52.6%)	-	0.208
Biologics, *n* (%)	-	7 (16.7%)	6 (31.6%)	-	0.318

*ACPA* anti-citrullinated peptide antibody, *BMI* body mass index, *CRP* C-reactive protein, *ESR* erythrocyte sedimentation rate, *DAS28* disease activity score in 28 joints, *HCQ* hydroxychloroquine, *MTX* methotrexate, *LEF* leflunomide, *RF* rheumatoid factor, *SR* sustained remission, *SSZ* sulfasalazine.

**Fig. 3:**
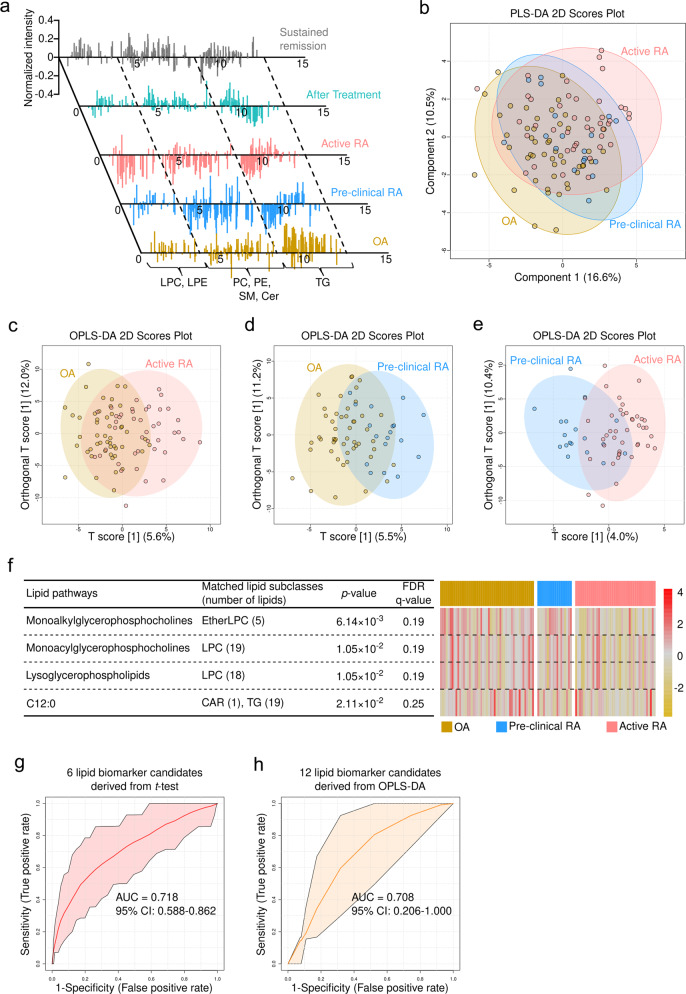
Comprehensive interpretation of the serum lipidome profile in RA patients according to disease phase and activity. **a** Normalized intensity of the identified lipids in representative samples belonging to five different groups (OA, preclinical RA, active RA (before treatment), paired samples after treatment, and sustained remission). **b** PLS-DA 2D score plots for the OA, preclinical RA, and active RA groups (*Q*^2^ = −0.016). OPLS-DA 2D score plots based on models of **c** OA and active RA, **d** OA and preclinical RA, and **e** preclinical RA and active RA (*Q*^2^ = 0.005, −0.090, and −0.363, respectively). **f** Expression of four significant lipid-related pathways shown as a heatmap; data were obtained from lipid ontology enrichment analysis of all lipids identified in the OA, preclinical RA, and active RA groups. **g** Six candidate lipid biomarkers (CAR 18:0, DG 36:2, LPC 16:1, LPC 18:1, LPC 20:1, and LPC O-16:1), identified by the *t* test (*p* value <0.05, FDR ≤ 0.25), which discriminate active RA from OA. **h** Twelve candidate lipid biomarkers (CAR 18:0, LPC 16:1, LPC 18:1, LPC 18:2, LPC 18:3, LPC 20:2, LPC 20:3, LPC 20:4, LPC 20:5, LPC 22:6, LPC O-18:0, and LPC O-18:1), identified by the OPLS-DA 2D score plot (|*r*| >0.5), which discriminate patients who go on to develop RA from patients with preclinical RA.

After data preprocessing and lipid identification, we obtained 238 individual lipids, which were assigned to 12 lipid subclasses. Of note, before statistical analysis, the normalized intensity of all identified lipids in serum samples from preclinical RA and active RA patients showed a similar pattern, whereas that in samples obtained from RA patients in sustained remission showed a pattern similar to that in the OA controls (Fig. 3a). Moreover, the expression of lipids in serum samples from active RA patients (prior to treatment) and paired samples obtained after treatment with DMARDs showed marked differences (Fig. 3a). Although we could not identify serum lipids that discriminated between preclinical RA, active RA, and OA (presumably due to overfitting of the PLS-DA model (*Q*^*2*^ of LOOCV was −0.016) caused by the high variation in clinical samples), the PLS-DA 2D score plots revealed that samples from preclinical RA patients showed a ‘transitional profile’ between OA and active RA (Fig. 3b). Among patients with RA, the lipidome profile did not differ according to ACPA- and/or RF-positivity.

To further explain the detailed differences between groups, we tried to conduct a pairwise comparison among groups using OPLS-DA. The results showed that the serum lipidome can discriminate between OA and active RA (*Q*^*2*^ of cross validation = 0.007) (Fig. 3c). In contrast, serum lipids did not distinguish OA from preclinical RA (*Q*^*2*^ of cross validation = −0.090) or preclinical RA from active RA (*Q*^*2*^ of cross validation = −0.363) (Fig. 3d, e). Twelve and six serum lipid candidates that discriminate active RA from OA were identified by OPLS-DA (|*r*| >0.5 was used as a cutoff value for selecting biomarker candidates) and a *t* test (*p* value <0.05 and FDR < 0.25), respectively (Fig. 3c and Table 3).

**Table 3:** Serum lipid biomarker candidates that discriminate active RA from OA.

	Lipid biomarker candidate	*p* value	FDR	Correlation coefficient (*r*)	Log_2_ (active RA/OA)
1	CAR 18:0	5.39 × 10^−^^3^	0.25	−0.51	−0.27
2	DG 36:2	9.90 × 10^−^^4^	0.24		−0.27
3	LPC 16:1	2.28 × 10^−^^3^	0.25	−0.59	−0.20
4	LPC 18:1	5.21 × 10^−^^3^	0.25	−0.59	−0.33
5	LPC 18:2			−0.54	−0.32
6	LPC 18:3			−0.62	−0.34
7	LPC 20:1	2.28 × 10^−^^3^	0.25		−0.27
8	LPC 20:2			−0.55	−0.29
9	LPC 20:3			−0.54	−0.22
10	LPC 20:4			−0.51	−0.26
11	LPC 20:5			−0.58	−0.25
12	LPC 22:6			−0.54	−0.18
13	LPC O-16:1	6.23 × 10^−^^3^	0.25		−0.21
14	LPC O-18:0			−0.61	−0.24
15	LPC O-18:1			−0.60	−0.24

The correlation coefficient (*r*) cutoff for candidate biomarkers was |*r*| >0.5. Additionally, lipids altered significantly in active RA compared with OA (*p* value <0.05; FDR < 0.25) were considered biomarker candidates. Lipid biomarker candidates identified by the *t* test are highlighted in red.

*CAR* acyl carnitine, *DG* diacylglycerol, *LPC* lysophosphatidylcholine, *LPC O-* ether-linked LPC.

To perform lipid ontology enrichment analysis, the intensity of the identified lipids was input into the analysis after normalization, and alterations in lipid metabolism according to RA phase were analyzed. This process identified four lipid pathways whose metabolism was altered significantly according to the phase of RA: monoalkylglycerophosphocholines, monoacylglycerophosphocholines, lysoglycerophospholipids, and C12:0. All were downregulated in patients with active RA compared with those with OA or preclinical RA (Fig. 3f; the list of individual lipids matched to each lipid pathway is shown in Supplementary Table 3).

### Diagnostic performance of selected serum lipid biomarker candidates

Based on data from the OPLS-DA and *t* test, we postulated that the serum lipid signature can discriminate active RA from OA. In particular, lipids showing significant differential expression in the serum of active RA patients might be potential biomarker candidates for discriminating active RA from OA (Table 3). To validate the performance of these potential lipid biomarker candidates, we carried out biomarker analysis (Supplementary Fig. 3a). The results showed that CAR 18:0, DG 36:2, LPC (16:1, 18:1, 20:1), and EtherLPC 16:1, differential expression of which was shown to be significant by the *t* test alone, exhibited the best ROC curve for biomarker analysis (AUC = 0.718; average accuracy based on 100 cross validations = 0.659) (Fig. 3g). Fifteen serum lipid biomarker candidates identified as significant by the *t* test and the OPLS-DA model also discriminated active RA from OA (AUC = 0.656; 95% confidence interval (CI), 0.503–0.782) (Supplementary Fig. 3b). Twelve lipid biomarker candidates identified by the OPLS-DA model alone showed relatively weak performance for discriminating active RA from OA (AUC = 0.583; *Q*^*2*^ = 0.086) (Supplementary Fig. 3c); however, they still showed a *Q*^*2*^ value (0.086) higher than that for all 238 identified lipids (0.005) (Supplementary Fig. 3a). Importantly, 12 lipids (CAR 18:0, LPC 16:1, LPC 18:1, LPC 18:2, LPC 18:3, LPC 20:2, LPC 20:3, LPC 20:4, LPC 20:5, LPC 22:6, LPC O-18:0, and LPC O-18:1) were also identified in 18 subjects at the preclinical phase of RA and differentiated seven patients who eventually progressed to definitive RA and fulfilled the RA classification criteria^6^ from the other 11 subjects who did not progress (AUC = 0.708; 95% CI, 0.206–1.000, Fig. 3h). These data suggest that lipids may be serum biomarkers that predict whether preclinical RA will progress to established RA.

**Fig. 4:**
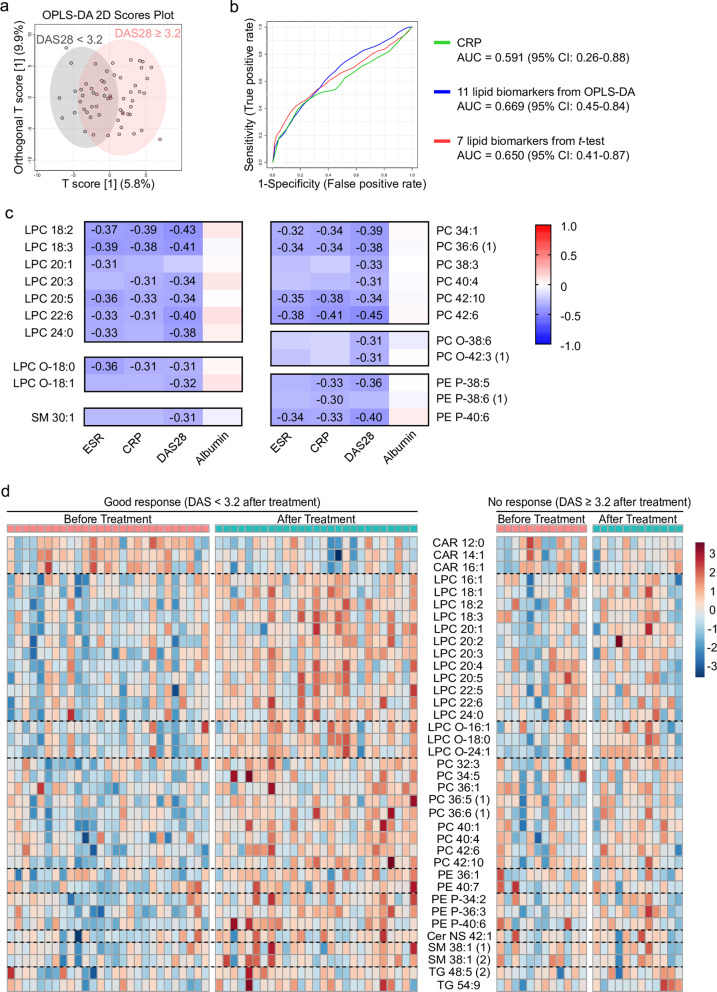
Diagnostic performance of candidate serum lipid biomarkers for assessing RA activity and treatment outcome. **a** OPLS-DA 2D score plots derived from models of moderate-to-high disease activity (DAS28 ≥ 3.2) and low disease activity or remission (DAS28 < 3.2) groups. **b** Validation of biomarker candidates from the discovery set identified by the *t* test (red) and OPLS-DA (blue). These biomarkers were used for biomarker analysis in the validation set. Area under the receiver operating characteristic (ROC) curve (AUC) analysis of the lipid biomarker candidates identified by the OPLS-DA and *t* test versus that for the C-reactive protein (as a comparator); parameters were evaluated for the ability to distinguish low disease activity from moderate/high disease activity, as determined by the DAS28 (green). The three compared areas are not significantly different. **c** Correlation between serum lipids and disease activity parameters. The exact value of the correlation coefficients is presented in a heatmap when absolute correlation coefficients are ≥0.3 (the *p* values for the correlation coefficient are <0.05). **d** Heatmap showing the expression of 37 significant lipids identified after comparison of paired samples obtained before and after treatment with DMARDs (samples compared using the *t* test).

Finally, we asked whether the serum lipidome reflects RA disease activity and whether it can predict treatment outcome. To this end, we pooled serum samples from patients with active RA and from those in sustained remission. The two groups were well discriminated by OPLS-DA (Fig. 4a). Thirteen serum lipid biomarker candidates (seven from the *t* test and 11 from the OPLS-DA model) were acquired (Table 4); all of these candidates were applied in a validation set to investigate whether they can differentiate moderate-to-high disease activity (DAS28 ≥ 3.2) from low disease activity or remission (DAS28 < 3.2). The AUC value of the ROC curve derived from seven biomarker candidates (LPC 18:2, LPC 18:3, LPC 20:3, LPC 22:6, LPC 24:0, PC 42:6, and SM 30:1) was 0.650, with an average accuracy of 0616 (calculated by 100 cross-validations) (Fig. 4b). Again, the AUC value for 11 lipids showing |*r*| >0.5 (i.e., LPC 18:0, LPC 18:2, LPC 18:3, LPC 20:0, LPC 20:2, LPC 20:3, LPC 20:4, LPC 20:5, LPC 22:6, LPC 24:0, and EtherPE 38:6 [2]) was 0.669, with an average accuracy of 0.615 (Fig. 4b). The AUCs for the seven or 11 lipid candidates were comparable with that for serum CRP (AUC = 0.591; *p* value = 0.765 vs. 11 lipids, and 0.850 vs. seven lipids; Fig. 4b), suggesting that these lipid candidates reflect RA disease activity comparable to CRP. Analysis of the correlation between RA activity parameters and individual serum lipids was also performed. The results showed that serum LPC subclasses, PC subclasses, EtherLPC (18:0 and 18:1), EtherPC (38:6 and 42:3 [1]), SM 30:1, and EtherPE (38:5, 38:6 [1], and 40:6) showed a negative correlation with markers of RA activity, such as the ESR, CRP, and DAS28 (Fig. 4c); such correlations were validated using follow-up serum samples from RA patients (*detailed results are available at Online Supplemental file 4*).

**Table 4:** Serum lipid biomarker candidates that discriminate moderate-to high disease activity (DAS28 ≥ 3.2) from low disease activity (DAS28 < 3.2).

	Lipid	*p* value	FDR	Correlation coefficient (*r*)	Log_2_ (DAS28 ≥ 3.2/DAS28 < 3.2)
1	LPC 18:0			−0.59	−0.27
2	LPC 18:2	1.12 × 10^−^^4^	2.65 × 10^−^^2^	−0.72	−0.64
3	LPC 18:3	3.24 × 10^−^^3^	1.80 × 10^−^^2^	−0.65	−0.50
4	LPC 20:0			−0.56	−0.27
5	LPC 20:2			−0.67	−0.45
6	LPC 20:3	3.79 × 10^−^^3^	1.80 × 10^−^^2^	−0.62	−0.42
7	LPC 20:4			−0.54	−0.35
8	LPC 20:5			−0.55	−0.28
9	LPC 22:6	1.10 × 10^−^^3^	8.68 × 10^−^^2^	−0.72	−0.54
10	LPC 24:0	3.87 × 10^−^^4^	4.58 × 10^−^^2^	−0.76	−0.56
11	PC 42:6	6.41 × 10^−^^3^	2.32 × 10^−^^1^		−0.36
12	PE P-38:6 (2)			−0.51	−0.53
13	SM 30:1	6.85 × 10^−^^3^	2.32 × 10^-1^		−0.39

The correlation coefficient (*r*) cutoff for biomarker candidates was |*r*| >0.5. Additionally, lipids altered significantly according to disease activity (*p* value <0.05; FDR < 0.25) were considered biomarker candidates.

*LPC* lysophosphatidylcholine, *PE P* ether-linked phosphatidylethanolamine, *SM* sphingomyelin.

All patients with active RA (DAS28 ≥ 3.2 before treatment) were monitored for 6 months after treatment with DMARDs. After 6 months, the patients could be divided into two groups according to treatment response: good responders (DAS28 < 3.2 after treatment) and nonresponders (DAS28 ≥ 3.2 after treatment). To compare changes in the serum lipidome profiles of the two groups, we conducted a paired *t* test and OPLS-DA using paired samples (pretreatment and posttreatment). In total, we noted a significant change in 41 serum lipids (37 lipids identified by the *t* test and 23 by the OPLS-DA; a detailed list of lipids is presented in Supplementary Table 4) in good responders after treatment, whereas no changes were observed in nonresponders (regularized Hotelling’s T^2^ [RHT] test^29^: good responders = 33.724, *p* value = 0.035; nonresponders = 6.294, *p* value = 1.000) (Supplementary Fig. 4a, b). Specifically, the serum LPC, EtherLPC, PC, PE, EtherPE, ceramide (Cer-NS) 42:1, SM, and TG subclasses increased, while the CAR subclass decreased, in good responders but not in nonresponders (Fig. 4d).

## Discussion

The current study demonstrates disease- and phase-dependent differences in the lipidome profiles in the SF and serum of RA patients compared with OA patients. In the SF, a variety of lipids were severely perturbed; these perturbations correlated well with the extent of joint inflammation, the severity of synovitis on ultrasonography, and systemic inflammatory markers (ESR and CRP). In the sera, the lipidome profile of active RA (albeit less prominent than the synovial lipidome) was also significantly distinct from that of RA in sustained remission; the profile in the latter was rather similar to that of patients with noninflammatory OA. Notably, the serum lipidome of patients with preclinical RA mimicked that of those with active RA. Moreover, alterations in LPC, PC, EtherPE, and/or SM subclasses correlated with RA activity as assessed by the DAS28 comparable to serum CRP and reflected treatment responses to DMARDs when monitored over time. Collectively, the data show that the lipidome profile in the SF and serum of RA patients is markedly perturbed. Such perturbations are noticeable at the preclinical phase before the disease progresses to definitive RA; these changes also reflect RA activity and treatment outcome.

The pathology of RA is characterized by synovial hyperplasia and massive infiltration by leukocytes. Here, global analysis of the lipidome clearly identified 135 synovial lipids associated with both RA itself and leukocytosis. Among these, the expression of 65 correlated well with the degree of inflammation and severity of synovitis on ultrasonography, suggesting that they may be a surrogate marker of synovial pathology, particularly the extent of synovial inflammation; thus, profiling these lipids may ameliorate the need for sonographic or pathologic examination of the synovia. With this specific notion in mind, we next investigated whether the synovial lipid fingerprint is also present in the systemic circulation. However, whereas 135 lipids were differentially expressed in the SF of RA patients, only 15 lipids in the serum of active RA patients showed significant alterations in expression. Moreover, whereas the expression patterns of LPC subclasses in serum and SF from active RA patients were similar, those of ether-linked lipids and ceramide were not. Therefore, arthritic joints and the systemic circulation appear to provide different microenvironments for lipid biogenesis and metabolism, although metabolic communication between the joint and the periphery cannot be excluded.

We believe that the most striking finding of our lipidome study is that changes in the lipidome are already established at the preclinical phase before the disease progresses. In general, lipids are utilized as substrates for the production of inflammatory mediators immediately after stimulation by cytokines, growth factors, and trauma^30^. Changes in lipid mediators can therefore be observed before disease manifestation^15,16^. For example, 5-hydroxyeicosatetraenoic acid (5-HETE) is elevated in the preclinical phase^16^. In addition, ω−3 fatty acid levels are decreased in pre-RA subjects with ACPA^31^, and short-chain carnitine levels are decreased in serum samples obtained prior to RA onset^32^. Our lipidome analysis demonstrates that lipid profiles in those with highly active RA with elevated ESR/CRP were similar to those in patients with preclinical RA and normal ESR/CRP, suggesting that alterations in lipid metabolism precede laboratory and clinical manifestations of RA. Of note, 12 serum lipids (CAR 18:0, LPC (16:1, 18:1, 18:2, 18:3, 20:2, 20:3, 20:4, 20:5, 22:6), and EtherLPC (18:0, 18:1)) differentiated patients who progressed to definite RA from those who did not. Given that ESR and CRP were normal in this preclinical RA subgroup, the lipid profile at the preclinical stage might be a more sensitive predictor of disease progression.

There is an unmet need with respect to assessing RA activity and treatment outcomes. For example, ESR and CRP are nonspecifically elevated by infection, and they can be normal in more than half of RA patients, regardless of disease activity^33,34^. Moreover, although DAS28 is widely used to assess RA, it must be calculated from the ESR or CRP value and assessed by experts^35^. Another noticeable finding of this study is that although deregulated serum lipids reflect disease activity, they can be restored after effective treatment; this trajectory is also evident in synovial lipids. For example, leukocyte-poor RA-SF showed a lipid pattern similar to OA-SF. Reduced expression of the serum LPC, PC, and SM subclasses correlated with RA activity assessed by the ESR, CRP, and DAS28. Moreover, serial monitoring of 41 serum lipids revealed significant alterations in good responders to treatment with DMARDs. Specifically, increases in the LPC, EtherLPC, PC, PE, EtherPE, Cer-NS 42:1, SM, and TG subclasses and a decrease in the CAR subclass were noted in good responders but not in nonresponders, which suggests that such changes represent treatment responses to DMARDs and thus might be an indicator for assessing treatment outcomes that may complement the ESR and CRP.

Endogenous lipids are the most important mediators of all phases of inflammation, as well as regulators of the inflammatory process (from initiation to cessation)^36^. They have been termed “bioactive lipids” and are divided into four main families according to their biochemical functions: classical eicosanoids, specialized pro-resolving mediators, lysoglycerophospholipids/sphingolipids, and endocannabinoids^37^. Lysoglycerophospholipids and sphingolipids comprise many compounds asymmetrically distributed in plasma membranes, with glycerol or sphingosine as backbones; these act as signaling molecules and play roles in cellular and tissue adaptation to inflammatory events (e.g., plasma membrane shaping, cell growth and death, and inflammatory cascades)^38^.

It is unclear how ‘lysoglycerophospholipids’ and ‘monoacylglycerophosphocholine’ pathways are downregulated in inflammatory RA-SF and in the serum of patients with active RA. LPC is synthesized by phospholipase A_2_ (PLA_2_) via hydrolysis of PC^39^ and is secreted mainly from the liver after stimulation by albumin^40^. The low concentration of LPC in active RA patients, therefore, might be due to a decrease in albumin and/or lipoprotein-associated PLA_2_ activity, which is common in such patients^41^. ‘Monoacylglycerophosphocholine’, an intermediate of the ‘glycerophosphocholine’ pathway, is also produced by hydrolysis of PC by PLA_2_^42^; thus, it can be compromised by reduced PLA_2_ activity.

The immunomodulatory effect of LPC is dependent on its biochemical structure; the proinflammatory effect is attributed to saturated and monounsaturated LPCs (LPC 16:0, LPC 18:0, LPC 18:1)^43^, whereas the anti-inflammatory effect is attributed to polyunsaturated LPC species (LPC 22:4, LPC 22:6)^44^. In this study, all serum LPCs, whether saturated, monounsaturated, or polyunsaturated, were decreased in active RA but increased during sustained remission; profiles in the SF showed a similar pattern. Interestingly, of all the lipidomes identified, LPC subclasses showed the most powerful negative correlation with RA activity, as well as the best response to DMARDs. Although the mechanism underlying the response to DMARDs remains elusive, we presume that it could be related to the ‘lipid paradox’, by which low-density lipoprotein (LDL) is sequestered in the liver under the high inflammatory conditions associated with RA^45^. Since LPC is a major component of oxidized LDL^39^, it can be increased after effective treatment with DMARDs. It is intriguing that LPC shows an inverse correlation with cardiovascular disease^46,47^, which is highly prevalent in RA patients^48^.

In contrast to LPC, PC subclasses, as well as components of the glycerophospholipid pathway, were overexpressed in RA-SF and showed a positive correlation with disease activity and synovitis severity; however, this was not seen in serum from patients with active RA. Similar contradictory expression of lipids between the SF and serum was also noted for ether-linked lipids and SM. Choline kinase, an enzyme essential for PC biosynthesis, is activated in RA synovial fibroblasts, thereby promoting cell migration and resistance to apoptosis^49^. Ether-linked lipids are synthesized by inflammatory stimuli and incorporated into the cellular membrane, where they act as cellular signaling molecules and antioxidants^50,51^. SM is involved in various pathophysiological functions, including apoptosis, autophagy, proliferation, differentiation, and invasiveness^37^. Ceramide, a catalytic product of SM, mediates TNFα-induced activation of NF-κB and regulates osteoclastogenesis via RANKL. Overall, dysregulated overexpression of PC, ether-linked lipids, and SM could be involved directly in the synovial pathology of RA; however, this does not seem to be mirrored in the systemic circulation.

This study has some limitations. First, the validation cohort was small. Although our major findings regarding the serum lipidome were reproduced consistently in RA patients who were monitored serially, further studies in a larger population of RA patients are needed to confirm the diagnostic performance of lipid biomarker candidates. Second, due to ethical and practical limitations of stopping DMARDs in patients with active RA, we cannot exclude the possible effects of medications on the lipidome profile. Notwithstanding this, we found no differences in the serum lipidome profile between active RA patients taking glucocorticoids and DMARDs and preclinical RA subjects who had never received them. Moreover, serum lipidome profiles were considerably different between patients with active RA and those in sustained remission, even though both groups received similar DMARDs. Therefore, we believe that anti-arthritis drugs, including nonsteroidal anti-inflammatory drugs, glucocorticoids, and DMARDs, do not greatly affect the lipidome profile in RA.

This study has several strengths. First, the phase of RA was rigorously ascertained; all biofluid samples were obtained in parallel with clinical and laboratory data, which were collected regularly from the cohort; these data included acute phase reactant levels and DAS28 scores. Thus, we provide evidence that not only RA itself but also the phase and status of disease at the time of sampling have a marked effect on the lipidome profile. Second, lipid biomarker candidates that discriminate different phases of disease activity were validated in serially monitored samples from the same patients; these samples were obtained and analyzed at different times during the disease course. Finally, we conducted ultrasonography simultaneously with arthrocentesis; the former allows examination of synovial pathology in real time and can determine synovitis severity and its correlation with the synovial lipidome. These data may strengthen the pathologic significance of the synovial lipidome identified in this study.

To the best of our knowledge, this is the first study to compare the lipidome profile at different phases of RA (preclinical, active, and sustained remission). The data suggest that the lipidome signature of active RA is lucid in the preclinical phase and that it changes with synovitis severity and inflammatory activity. We believe that knowledge of the lipid profile of patients in the preclinical, active, and sustained remission phases sheds light on the lipid metabolic pathways involved in RA initiation, perpetuation, and resolution. Moreover, the lipid biomarker candidates suggested in this study may be clinically useful for the early identification of individuals at risk of evolution to definitive RA and for establishing a therapeutic strategy based on disease activity. We envisage that the determination of lipidome signatures could form part of a precision medicine approach to treating RA and propose that these potential biomarker candidates should be validated in further large-scale clinical studies.

## Supplementary information

Supplementary Information
