# Supplementary material for: Lipidome profile predictive of disease evolution and activity in rheumatoid arthritis
Source: Exp Mol Med. 2022 Feb 15;54(2):143–55. doi: 10.1038/s12276-022-00725-z (PMC8894401; doi:10.1038/s12276-022-00725-z)
Supplement: Supplementary file 1 — Supplementary Information [file 12276_2022_725_MOESM1_ESM.pdf]

Supplementary Information

Lipidome profile predictive of disease evolution and activity in rheumatoid arthritis

Jung Hee Koh[1,2*], Sang Jun Yoon[3*], Mina Kim[3], Seonghun Cho[4], Johan Lim[4], Youngjae Park[1], Hyun-Sook Kim[5], Sung Won Kwon[3†], and Wan-Uk Kim[1,2†]

[1]Division of Rheumatology, Department of Internal Medicine, the Catholic University of Korea, Seoul 06591, Korea

[2]Center for Integrative Rheumatoid Transcriptomics and Dynamics, the Catholic University of Korea, Seoul 06591, Korea

[3]College of Pharmacy, Seoul National University, Seoul 08826, Republic of Korea

[4]Department of Statistics, Seoul National University, Seoul 08826, Republic of Korea

[5]Department of Internal Medicine, Soonchunhyang University College of Medicine, Seoul 04401, Republic of Korea

[*] These authors contributed equally to this work.

[†]Correspondence and reprint requests to:

Professor Wan-Uk Kim, M.D., Ph.D. Division of Rheumatology, Department of Internal Medicine, Seoul St. Mary's Hospital, Center for Integrative Rheumatoid Transcriptomics and Dynamics, College of Medicine, The Catholic University of Korea, 222 Banpo-daero, Seocho-gu, Seoul 08826, Republic of Korea. E-mail: wan725@catholic.ac.kr; Tel.: +82-2-2258-7530; Fax: +82-2-2258-7526

Or

Professor Sung Won Kwon, Ph.D. College of Pharmacy, Seoul National University, 1 Gwanak-ro, Gwanak-gu, Seoul 08826, Republic of Korea. E-mail: swkwon@snu.ac.kr, Tel.: +82-2-880-7844; Fax: +82-2-886-7844

This file includes:

Supplementary Fig. 1. Venn diagram showing how RA and leukocytosis alter the lipid profile in synovial fluid (SF).

Supplementary Fig. 2. Multivariate exploratory ROC analysis using Monte-Carlo cross validation (MCCV) to predict synovitis severity.

Supplementary Fig. 3. Cross validation of the OPLS-DA models, and ROC curves for serum lipid biomarker candidates that differentiate active RA from OA.

Supplementary Fig. 4. Approximate null distributions derived from the Regularized Hotelling's T^2 test.

Supplementary Table 1. Baseline characteristics of synovial fluid donors

Supplementary Table 2. Lipid ontology enrichment analysis for synovial lipid metabolism

Supplementary Table 3. Lipid ontology analysis of the serum lipidome enriched in active RA

Supplementary Table 4. Lipidomes showing altered expression after treatment with anti-rheumatic drugs

---

Supplementary file 1. List of all identified lipids in leukocyte-rich rheumatoid arthritis (RA)-synovial fluid (SF), leukocyte-poor RA-SF, and osteoarthritis (OA)-SF groups.

Supplementary file 2. Differences in lipidome profiles between rheumatoid arthritis (RA)-synovial fluid (SF) and osteoarthritis (OA)-SF, and between leukocyte-rich and leukocyte-poor SF.

Supplementary file 3. Synovial lipidome predictive of synovitis severity on ultrasonography.

Supplementary file 4. Correlation between the lipidome and RA activity in sera collected before and after treatment with DMARDs.

*Supplementary files 1–4 are available at https://www.cirad-catholic.com/supplementary-figures-data

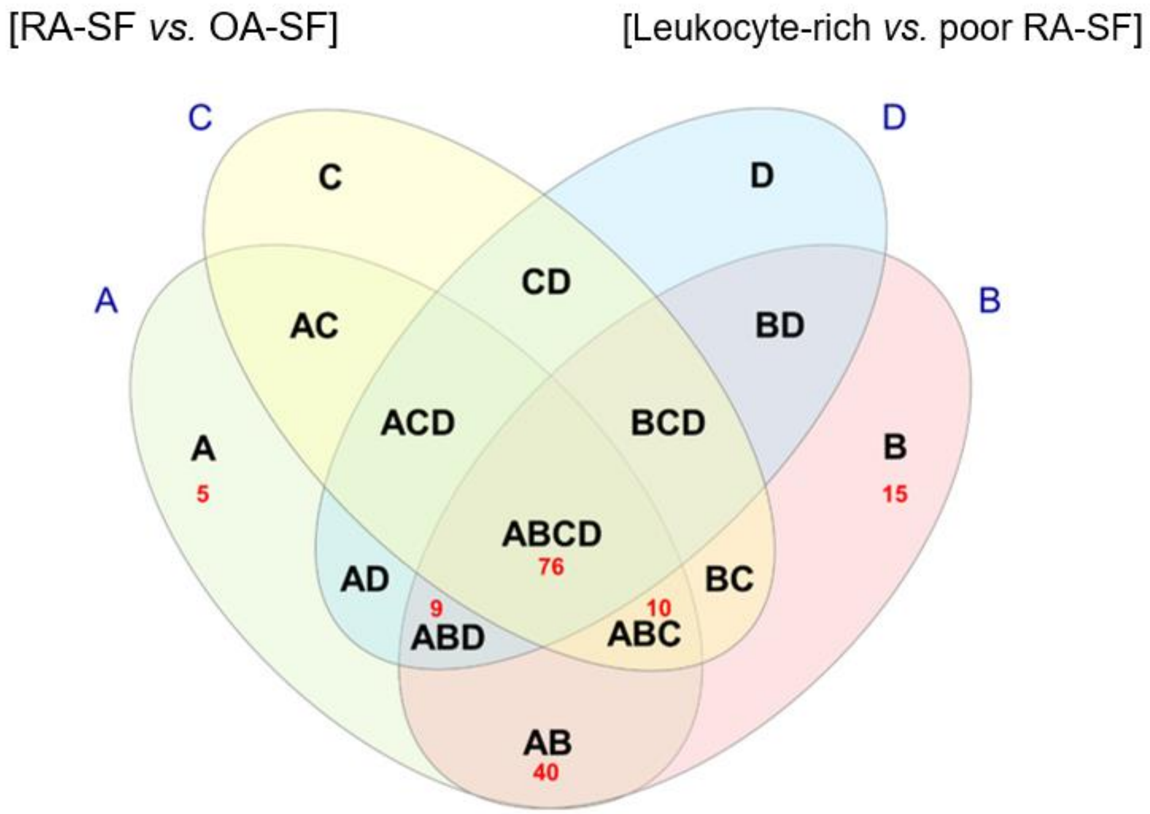

Supplementary Fig. 1. Venn diagram showing how RA and leukocytosis alter the lipid profile in synovial fluid (SF). **A**: Significant differences in the synovial lipidome between RA-SF and osteoarthritis (OA)-SF, as identified by the *t*-test. **B**: Significant differences in the synovial lipidome between leukocyte-rich SF and leukocyte-poor SF (which combines leukocyte-poor RA-SF and OA-SF) groups, as identified by the *t*-test. **C**: Synovial lipidome with variable importance for a projection (VIP) score >1 in the PLS-DA model based on RA-SF and OA-SF. **D**: Synovial lipidome with a VIP score >1 in the PLS-DA model based on leukocyte-rich SF and leukocyte-poor SF. The number of overlapping lipids is highlighted in red.

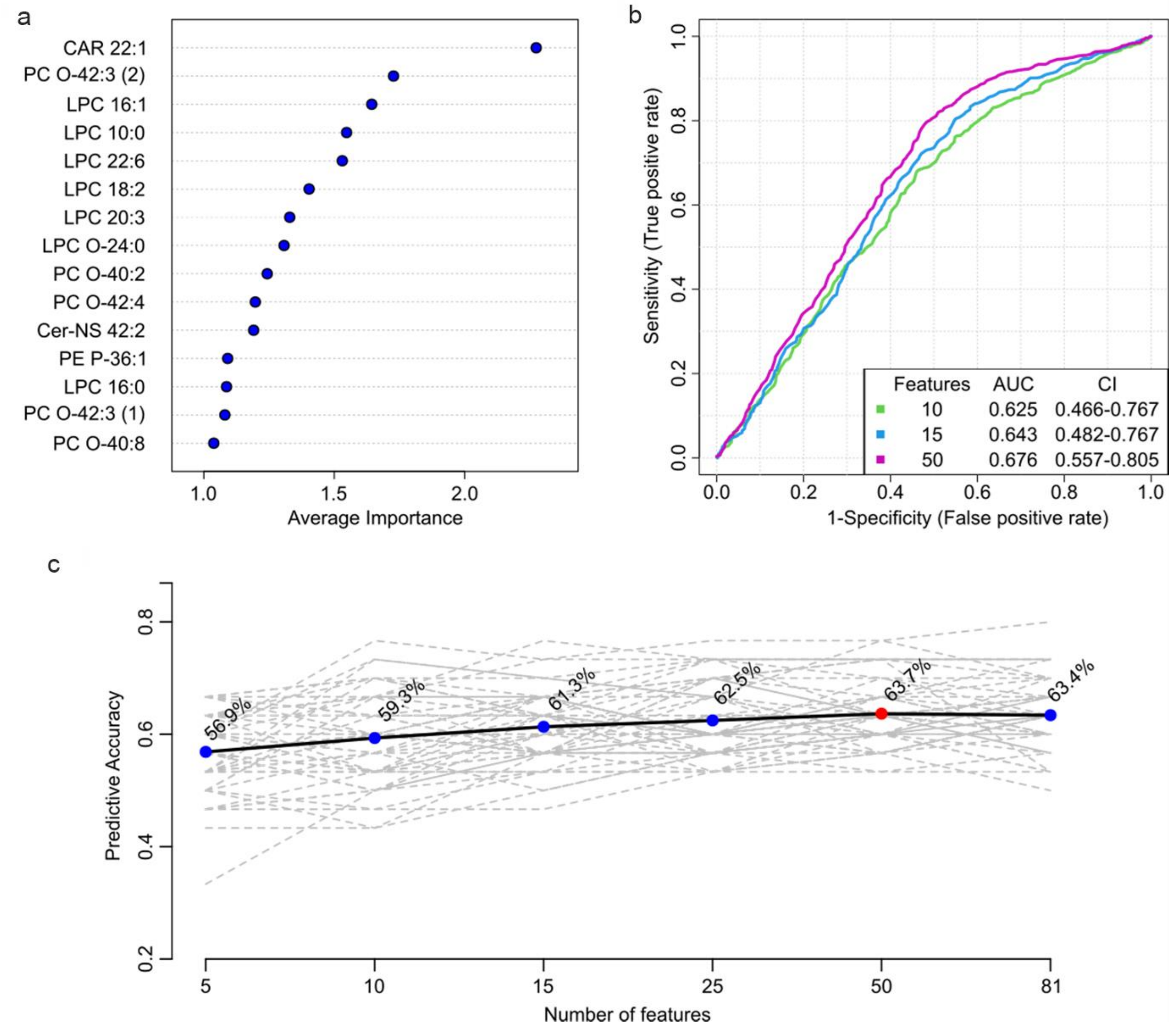

Supplementary Fig. 2. Multivariate exploratory ROC analysis using Monte-Carlo cross validation (MCCV) to predict synovitis severity. (a) All identified synovial lipids were examined, and the importance of features for predicting synovitis severity (moderate-to-severe *vs*. mild synovitis) was evaluated using 2/3 of samples per MCCV. **(b)** The AUC of the ROC according to feature number, based on Random Forests classification. **(c)** Each model was validated using the remaining 1/3 samples, and predictive accuracy was assessed.

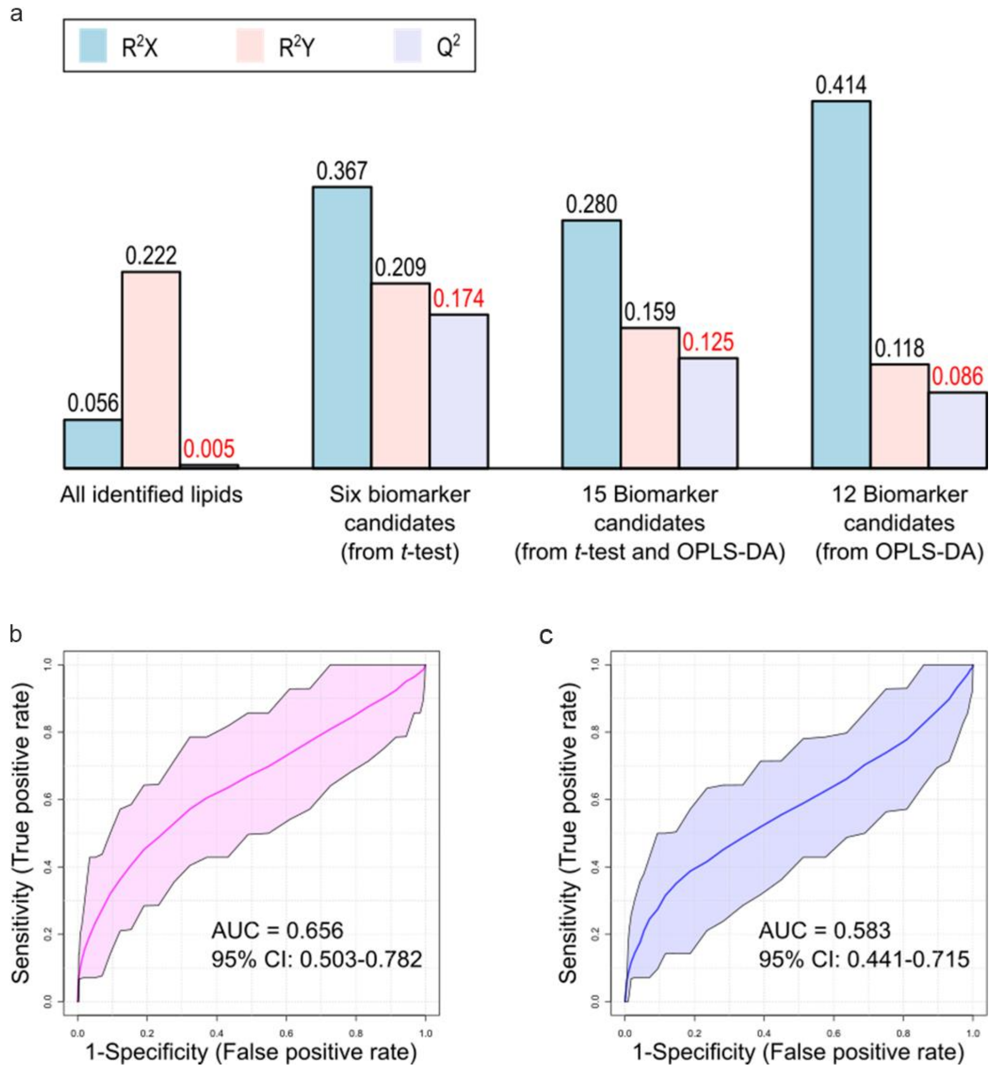

Supplementary Fig. 3. Cross validation of the OPLS-DA models, and ROC curves for serum lipid biomarker candidates that differentiate active RA from OA. (a) Cross validation of OPLS-DA models based on differential lipid composition. Q^2 shows the best discriminatory performance when the OPLS-DA model was established using six biomarker candidates (CAR 18:0, DG 36:2, LPC 16:1, LPC 18:1, LPC 20:1, and LPC O-16:1) identified by the *t*-test. **(b)** ROC curve for 15 biomarker candidates identified by the *t*-test and the OPLS-DA model. The AUC of the ROC curve was 0.656, and the average accuracy based on 100 cross validations was 0.621. **(c)** ROC curve for 12 biomarker candidates (CAR 18:0, LPC 16:1, LPC 18:1, LPC 18:2, LPC 18:3, LPC 20:2, LPC 20:3, LPC 20:4, LPC 20:5, LPC 22:6, LPC O-18:0, and LPC O-18:1) identified by the OPLS-DA model. The AUC of the ROC curve was 0.583, and the average accuracy based on 100 cross validations was 0.559.

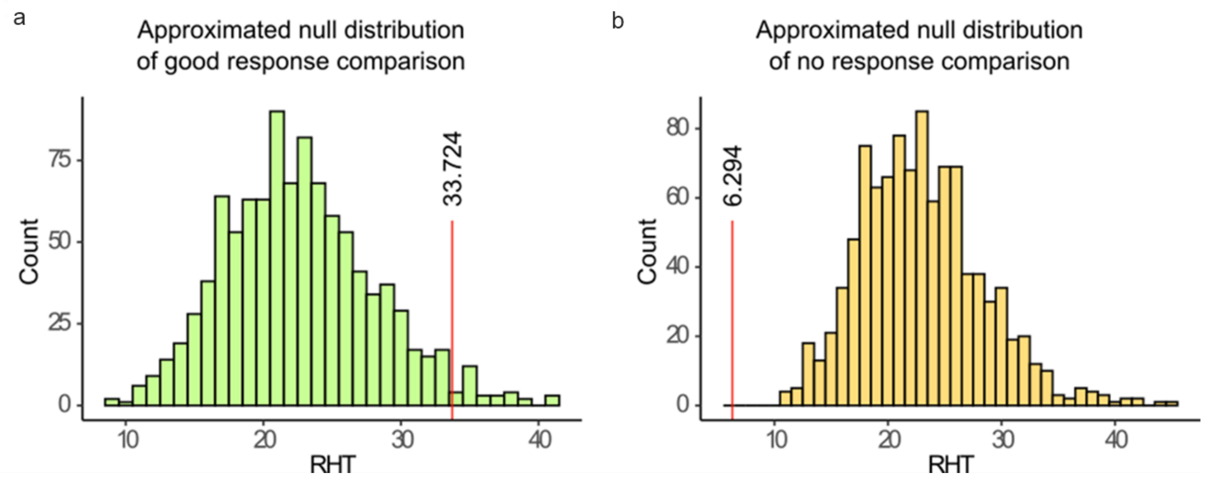

Supplementary Fig. 4. Approximate null distributions derived from the Regularized Hotelling's T^2 test. The Regularized Hotelling's T^2 (RHT) test was used to estimate differences in expression of 41 lipids in each comparison set (good response and non-response) before and after treatment. **(a)** The test statistic and the *p*-value for the good response comparison set were 33.724 and 0.035, respectively. **(b)** The values for the non-response set were 6.294 and 1.000, respectively. The count indicates the RHT values derived from an approximated null distribution.

Supplementary Table 1. Baseline characteristics of synovial fluid donors

**Synovial fluid donors**	Leukocyte-rich RA-SF (n = 55)	Leukocyte-poor RA-SF (n = 16)	OA-SF (n = 31)	*p*-value
Age, years	59.3 ± 14.9	64.6 ± 8.8	63.8 ± 10.6	0.357
Female, n (%)	49 (87.5)	14 (82.4)	26 (83.9)	0.825
Disease duration, years	1 (1–3)	1 (1–3.5)	-	0.924
RF-positive, n (%)	41/52 (78.9)	13/17 (76.5)	-	0.837
ACPA-positive, n (%)	36/44 (81.8)	11/12 (91.7)	-	0.410
Laboratory findings				
ESR, mm/h	29.6 ± 22.4	11.8 ± 11.2	-	0.003
CRP, mg/dL	2.5 ± 3.2	0.3 ± 0.5	-	<0.001
SF-WBC count, /μL	15,535 ± 14,355	979 ± 890.8	-	<0.001
MTX, n (%)	31 (56.4)	9 (56.3)	-	0.816
LEF, n (%)	15 (27.3)	6 (37.5)	-	0.306
HCQ, n (%)	18 (32.7)	4 (25.0)	-	0.443
SSZ, n (%)	13 (23.6)	1 (6.3)	-	0.166
Biologics, n (%)	15 (27.3)	5 (31.3)		0.544

ACPA, anti-citrullinated protein antibody; CRP, C-reactive protein; ESR, erythrocyte sedimentation rate; HCQ, hydroxychloroquine; MTX, methotrexate; LEF, leflunomide; OA-SF, synovial fluid from osteoarthritis patients; RA-SF, synovial fluid from rheumatoid arthritis patients; RF, rheumatoid factor; SSZ, sulfasalazine; SF-WBC, synovial fluid white blood cell.

Supplementary Table 2. Lipid ontology enrichment analysis for synovial lipid metabolism

Lipid pathways	Matched individual lipids	*p*-value	FDR
Lysoglycerophospholipids	LPC 10:0, LPC 14:0, LPC 16:0, LPC 16:1, LPC 18:0, LPC 18:1, LPC 18:2, LPC 18:3, LPC 20:0, LPC 20:1, LPC 20:2, LPC 20:3, LPC 20:4, LPC 20:5, LPC 22:5, LPC 22:6, LPC 24:0, LPC 24:6, LPC 26:1, LPE 16:0	2.40×10^{-6}	1.02×10^{-4}
Monoacylglycerophosphocholines	LPC 10:0, LPC 14:0, LPC 16:0, LPC 16:1, LPC 18:0, LPC 18:1, LPC 18:2, LPC 18:3, LPC 20:0, LPC 20:1, LPC 20:2, LPC 20:3, LPC 20:4, LPC 20:5, LPC 22:5, LPC 22:6, LPC 24:0, LPC 24:6, LPC 26:1	7.80×10^{-6}	1.33×10^{-4}
1-alkyl,2-acylglycerophosphocholines	PC O-32:1, PC O-34:4, PC O-36:1, PC O-36:4 (1), PC O-36:4 (2), PC O-36:5 (1), PC O-36:5 (2), PC O-38:3, PC O-38:4, PC O-38:5 (1), PC O-38:5 (2), PC O-38:6, PC O-38:7, PC O-40:1, PC O-40:2, PC O-40:3, PC O-40:5, PC O-40:6, PC O-40:8, PC O-42:3 (1), PC O-42:3 (2), PC O-42:4	7.30×10^{-6}	1.33×10^{-4}
Contains ether-bond	PC O-32:1, PC O-34:4, PC O-36:1, PC O-36:4 (1), PC O-36:4 (2), PC O-36:5 (1), PC O-36:5 (2), PC O-38:3, PC O-38:4, PC O-38:5 (1), PC O-38:5 (2), PC O-38:6, PC O-38:7, PC O-40:1, PC O-40:2, PC O-40:3, PC O-40:5, PC O-40:6, PC O-40:8, PC O-42:3 (1), PC O-42:3 (2), PC O-42:4	7.30×10^{-6}	1.33×10^{-4}
Glycerophosphocholines	LPC 10:0, LPC 14:0, LPC 16:0, LPC 16:1, LPC 18:0, LPC 18:1, LPC 18:2, LPC 18:3, LPC 20:0, LPC 20:1, LPC 20:2, LPC 20:3, LPC 20:4, LPC 20:5, LPC 22:5, LPC 22:6, LPC 24:0, LPC 24:6, LPC 26:1, LPC O-16:0, LPC O-16:1, LPC O-18:0, LPC O-18:3, LPC O-22:0, LPC O-22:1, LPC O-24:0, LPC O-24:1, PC 24:0, PC 28:0 (1), PC 28:0 (2), PC 30:1 (1), PC 30:1 (2), PC 30:2, PC 32:0, PC 32:1, PC 32:2, PC 32:3, PC 34:0, PC 34:1, PC 34:2, PC 34:3, PC 34:4, PC 34:5, PC 36:2, PC 36:3, PC 36:4 (1), PC 36:4 (2), PC 36:5 (1), PC 36:5 (2), PC 36:6 (1), PC 36:6 (2), PC 38:2, PC 38:3, PC 38:5 (1), PC 38:5 (2), PC 38:6 (1), PC 38:6 (2), PC 38:7, PC 40:2, PC 40:4, PC 40:5, PC 40:6, PC 40:8, PC 42:10, PC 42:4, PC 42:7, PC 42:8, PC 44:10, PC O-32:1, PC O-34:4, PC O-36:1, PC O-36:4 (1), PC O-36:4 (2), PC O-36:5 (1), PC O-36:5 (2), PC O-38:3, PC O-38:4, PC O-38:5 (1), PC O-38:5 (2), PC O-38:6, PC O-38:7, PC O-40:1, PC O-40:2, PC O-40:3, PC O-40:5, PC O-40:6, PC O-40:8, PC O-42:3 (1), PC O-42:3 (2), PC O-42:4	2.50×10^{-4}	3.04×10^{-3}
Glycerophospholipids	LPC 10:0, LPC 14:0, LPC 16:0, LPC 16:1, LPC 18:0, LPC 18:1, LPC 18:2, LPC 18:3, LPC 20:0, LPC 20:1, LPC 20:2, LPC 20:3, LPC 20:4, LPC 20:5, LPC 22:5, LPC 22:6, LPC 24:0, LPC 24:6, LPC 26:1, LPC O-16:0, LPC O-16:1, LPC O-18:0, LPC O-18:3, LPC O-22:0, LPC O-22:1, LPC O-24:0, LPC O-24:1, PC 24:0, PC 28:0 (1), PC 28:0 (2), PC 30:1 (1), PC 30:1 (2), PC 30:2, PC 32:0, PC 32:1, PC 32:2, PC 32:3, PC 34:0, PC 34:1, PC 34:2, PC 34:3, PC 34:4, PC 34:5, PC 36:2, PC 36:3, PC 36:4 (1), PC 36:4 (2), PC 36:5 (1), PC 36:5 (2), PC 36:6 (1), PC 36:6 (2), PC 38:2, PC 38:3, PC 38:5 (1), PC 38:5 (2), PC 38:6 (1), PC 38:6 (2),	9.76×10^{-3}	7.54×10^{-2}

	PC 38:7, PC 40:2, PC 40:4, PC 40:5, PC 40:6, PC 40:8, PC 42:10, PC 42:4, PC 42:7, PC 42:8, PC 44:10, PC O-32:1, PC O-34:4, PC O-36:1, PC O-36:4 (1), PC O-36:4 (2), PC O-36:5 (1), PC O-36:5 (2), PC O-38:3, PC O-38:4, PC O-38:5 (1), PC O-38:5 (2), PC O-38:6, PC O-38:7, PC O-40:1, PC O-40:2, PC O-40:3, PC O-40:5, PC O-40:6, PC O-40:8, PC O-42:3 (1), PC O-42:3 (2), PC O-42:4, LPE 16:0, LPE O-18:1, PE 36:5, PE 38:4, PE 38:6, PE 40:6, PE P-34:1, PE P-36:1, PE P-36:2, PE P-36:3, PE P-36:5, PE P-38:4, PE P-38:5 (1), PE P-38:5 (2)

Abbreviations: LPC, lysophosphatidylcholine; LPC-O, ether-linked lysophosphatidylcholine; PC, phosphatidylcholine; PC-O, ether-linked phosphatidylcholine; PE, phosphatidylethanolamine; PE P, ether-linked phosphatidylethanolamine.

Supplementary Table 3. Lipid ontology analysis of serum lipidome enriched by active RA

Lipid pathways*	Matched individual lipid	*p*-value	FDR
Monoalkylglycerophosphocholines	LPC O-16:0, LPC O-16:1, LPC O-18:0 LPC O-18:1, LPC O-24:1	6.14×10^{-3}	0.19
Monoacylglycerophosphocholines	LPC 14:0, LPC 16:0, LPC 16:1, LPC 18:0, LPC 18:1, LPC 18:2, LPC 18:3, LPC 20:0, LPC 20:1, LPC 20:2, LPC 20:3, LPC 20:4, LPC 20:5, LPC 22:1, LPC 22:4, LPC 22:5, LPC 22:6, LPC 24:0	1.05×10^{-2}	0.19
Lysoglycerophospholipids	LPC 14:0, LPC 16:0, LPC 16:1, LPC 18:0, LPC 18:1, LPC 18:2, LPC 18:3, LPC 20:0, LPC 20:1, LPC 20:2, LPC 20:3, LPC 20:4, LPC 20:5, LPC 22:1, LPC 22:4, LPC 22:5, LPC 22:6, LPC 24:0	1.05×10^{-2}	0.19
C12:0	CAR 12:0, TG 36:0, TG 40:0, TG 40:1, TG 42:0, TG 42:1 (2), TG 42:2 (2), TG 44:1 (1), TG 44:1 (2), TG 44:2, TG 44:3, TG 46:1, TG 46:2, TG 46:3 (1), TG 46:3 (2), TG 46:4 (2), TG 48:3, TG 48:5 (1), TG 50:6 (1), TG 50:7	2.11×10^{-2}	0.25

*The normalized intensity of identified serum lipids was used to evaluate whether lipid metabolism was altered in the active RA subgroup compared with the OA or pre-RA subgroups.

Abbreviations: CAR, acyl carnitine; LPC, lysophosphatidylcholine; LPC-O, ether-linked LPC; TG, triacylglycerol.

Supplementary Table 4. Lipidomes showing altered expression after treatment with anti-rheumatic drugs

	Biomarker candidate	*p*-value	FDR	Correlation coefficient (*r*)	Log_2 (after/before treatment)
1	CAR 12:0	2.13×10^{-3}	3.89×10^{-2}		-0.32
2	CAR 14:1	2.16×10^{-2}	1.79×10^{-1}		-0.64
3	CAR 16:1	1.15×10^{-2}	1.24×10^{-1}		-0.47
4	Cer-NS 42:1	3.14×10^{-2}	2.12×10^{-1}		0.26
5	LPC 16:0			0.52	0.26
6	LPC 16:1	1.35×10^{-3}	2.76×10^{-2}	0.57	0.38
7	LPC 18:1	6.67×10^{-3}	7.93×10^{-2}	0.64	0.46
8	LPC 18:2	2.51×10^{-4}	1.63×10^{-2}	0.63	0.70
9	LPC 18:3	1.62×10^{-4}	1.63×10^{-2}	0.70	0.89
10	LPC 20:1	1.24×10^{-3}	2.76×10^{-2}		0.36
11	LPC 20:2	3.78×10^{-3}	5.29×10^{-2}	0.55	0.42
12	LPC 20:3	3.82×10^{-4}	1.63×10^{-2}	0.63	0.61
13	LPC 20:4	7.17×10^{-3}	8.12×10^{-2}	0.52	0.49
14	LPC 20:5	6.12×10^{-3}	7.66×10^{-2}	0.52	0.49
15	LPC 22:5	2.88×10^{-3}	4.72×10^{-2}	0.54	0.54
16	LPC 22:6	3.07×10^{-3}	4.72×10^{-2}	0.55	0.54
17	LPC 24:0	6.04×10^{-3}	7.66×10^{-2}		0.43
18	LPC O-16:1	1.76×10^{-2}	1.67×10^{-1}		0.28
19	LPC O-18:0	4.78×10^{-4}	1.63×10^{-2}	0.65	0.51
20	LPC O-24:1	1.30×10^{-2}	1.31×10^{-1}		0.37
21	PC 32:3	2.45×10^{-2}	1.83×10^{-1}		0.41
22	PC 34:2			0.50	0.24
23	PC 34:5	3.17×10^{-3}	4.72×10^{-2}	0.57	0.57
24	PC 36:1	2.56×10^{-2}	1.85×10^{-1}		0.25
25	PC 36:5 (1)	2.17×10^{-2}	1.79×10^{-1}		0.26
26	PC 36:6 (1)	4.43×10^{-4}	1.63×10^{-2}	0.68	0.76
27	PC 40:1	3.30×10^{-4}	1.63×10^{-2}	0.77	0.89
28	PC 40:4	1.32×10^{-2}	1.31×10^{-1}		0.46
29	PC 42:10	1.91×10^{-4}	1.63×10^{-2}	0.67	0.47
30	PC 42:6	1.39×10^{-3}	2.76×10^{-2}	0.62	0.46
31	PC O-36:3			0.53	0.27
32	PE 36:1	2.47×10^{-2}	1.83×10^{-1}		0.35
33	PE 40:7	1.82×10^{-2}	1.67×10^{-1}		0.33
34	PE P-34:2	3.07×10^{-2}	2.12×10^{-1}		0.31
35	PE P-36:3	8.06×10^{-4}	2.40×10^{-2}	0.66	0.50

36	PE P-40:6	9.35×10^{-4}	2.47×10^{-2}	0.59	0.56
37	SM 38:1 (1)	2.18×10^{-2}	1.79×10^{-1}		0.22
38	SM 38:1 (2)	2.39×10^{-2}	1.83×10^{-1}		0.27
39	TG 48:5 (2)	3.70×10^{-2}	2.38×10^{-1}		0.83
40	TG 50:6 (4)			0.54	0.68
41	TG 54:9	3.21×10^{-2}	2.12×10^{-1}	0.52	0.81

The list of lipid biomarker candidates was detected by OPLS-DA based on pre- and post-treatment samples obtained from good responders. The *t*-test was used to identify additional biomarker candidates showing significant alteration in expression after treatment with DMARDs. The correlation coefficient (*r*) cut-off for biomarker candidates was $|r| > 0.5$. Lipids altered significantly by disease activity (p-value <0.05, FDR <0.25) were also considered as biomarker candidates.

Abbreviations: CAR, acyl carnitine; Cer, ceramide; LPC, lysophosphatidylcholine; LPC O, ether-linked LPC; PC, phosphatidylcholine; PE, phosphatidylethanolamine; PE P, ether-linked PE; TG, triacylglycerol.
